# Pathophysiology and management of risperidone-induced sialorrhea: case report

**DOI:** 10.3389/fpsyt.2023.1185750

**Published:** 2023-07-13

**Authors:** Tyler Torrico, Angad Kahlon

**Affiliations:** ^1^Kern Medical, Department of Psychiatry, Bakersfield, CA, United States; ^2^American Psychiatric Association Substance Abuse and Mental Health Services Administration (SAMHSA) Minority Fellowship, Washington, DC, United States

**Keywords:** antipsychotics, adverse reactions, psychopharmacology, second-generation antipsychotics, schizophrenia, iloperidone

## Abstract

**Background:**

Among antipsychotics, sialorrhea is most associated with clozapine, and when it occurs, it is uncomfortable, socially stigmatizing, and can contribute to medication non-adherence. Risperidone has a generally negligible muscarinic activity compared to clozapine, and yet, multiple reports of severe sialorrhea associated with risperidone have been reported.

**Case presentation:**

This case report describes risperidone-induced sialorrhea that was unintentionally masked by simultaneous clonidine administration that was intended to treat hypertension. Interestingly, sialorrhea was present but mild when clonidine was present; however, when risperidone was further titrated and clonidine removed, a significant worsening of sialorrhea developed. Sialorrhea did not respond to treatment with anticholinergic medication.

**Conclusion:**

The pathophysiology of antipsychotic-induced sialorrhea is complex and varies between antipsychotics. Risperidone-induced sialorrhea is suspected of having prominent adrenergic pathophysiology that is likely composed of highly viscoelastic saliva (high protein content), differing from the more commonly encountered clozapine-induced sialorrhea. Risperidone-induced sialorrhea is reported as more likely to respond to dose reduction and treatment with α2-adrenergic receptor agonists or β-adrenergic receptor antagonists and less likely to respond to anticholinergic (antimuscarinic) medications.

## Background

Sialorrhea is a known potential adverse reaction of antipsychotic medications; however, its incidence varies among antipsychotics, and its pathophysiology is not unanimous. Salivary flow is predominantly under parasympathetic (cholinergic) control, but the sympathetic (adrenergic) system also modulates saliva production (1). Among antipsychotics, sialorrhea is most associated with clozapine, and when it occurs, it is uncomfortable, socially stigmatizing, and can contribute to medication non-adherence (2). Clozapine (including metabolites) has a generally high muscarinic activity compared to the other antipsychotics, which significantly contributes to sialorrhea when it occurs. In contrast, risperidone has a generally negligible muscarinic activity; and yet, multiple reports of severe sialorrhea associated with risperidone have been reported (3–6). This difference demonstrates that sialorrhea as an adverse reaction of antipsychotics has a complex pathophysiology and that the saliva itself in antipsychotic-associated sialorrhea may have different make-up depending on cholinergic vs. adrenergic stimulation proportions. We present a case of risperidone-induced sialorrhea masked by clonidine that demonstrates risperidone's adrenergic properties having a significant contribution to saliva overproduction.

## Case report

A 46-year-old Caucasian male with a medical history of hypertension and psychiatric history of schizoaffective disorder, depressed type, presented to the behavioral health unit with paranoid ideation and aggression. He had previously tolerated quetiapine at 800 mg but was medication non-adherent for an unknown duration of time prior to this presentation. On admission, his vitals were normal except for hypertension (154/94 mmHg). Urine toxicology screening was negative, and his labs were unremarkable. On hospital day 1, he was started on risperidone 1 mg twice daily to treat psychosis. On hospital day 2, risperidone was increased to 1.5 mg twice daily. Simultaneously, the patient was started on clonidine 0.1 mg twice daily for ongoing hypertension. Clonidine was chosen for short-term use, as it was unclear if the patient had chronic hypertension in early hospitalization. As hypertension continued, on hospital day 3, amlodipine 10 mg daily was added to his regimen with titration of risperidone to 2 mg twice daily. On hospital day 4, the patient exhibited mild sialorrhea [drooling severity score: 3, moderate (2, 7, 8)] along with mild muscle rigidity and was administered benztropine 2 mg intramuscular for extrapyramidal symptoms that alleviated the rigidity only. Throughout that night, his blood pressure remained elevated, prompting consultation with internal medicine, who recommended discontinuing his clonidine and adding lisinopril 20 mg daily. The final hypertension regimen for hospitalization was lisinopril 20 mg daily and amlodipine 10 mg daily.

**Figure 1:**
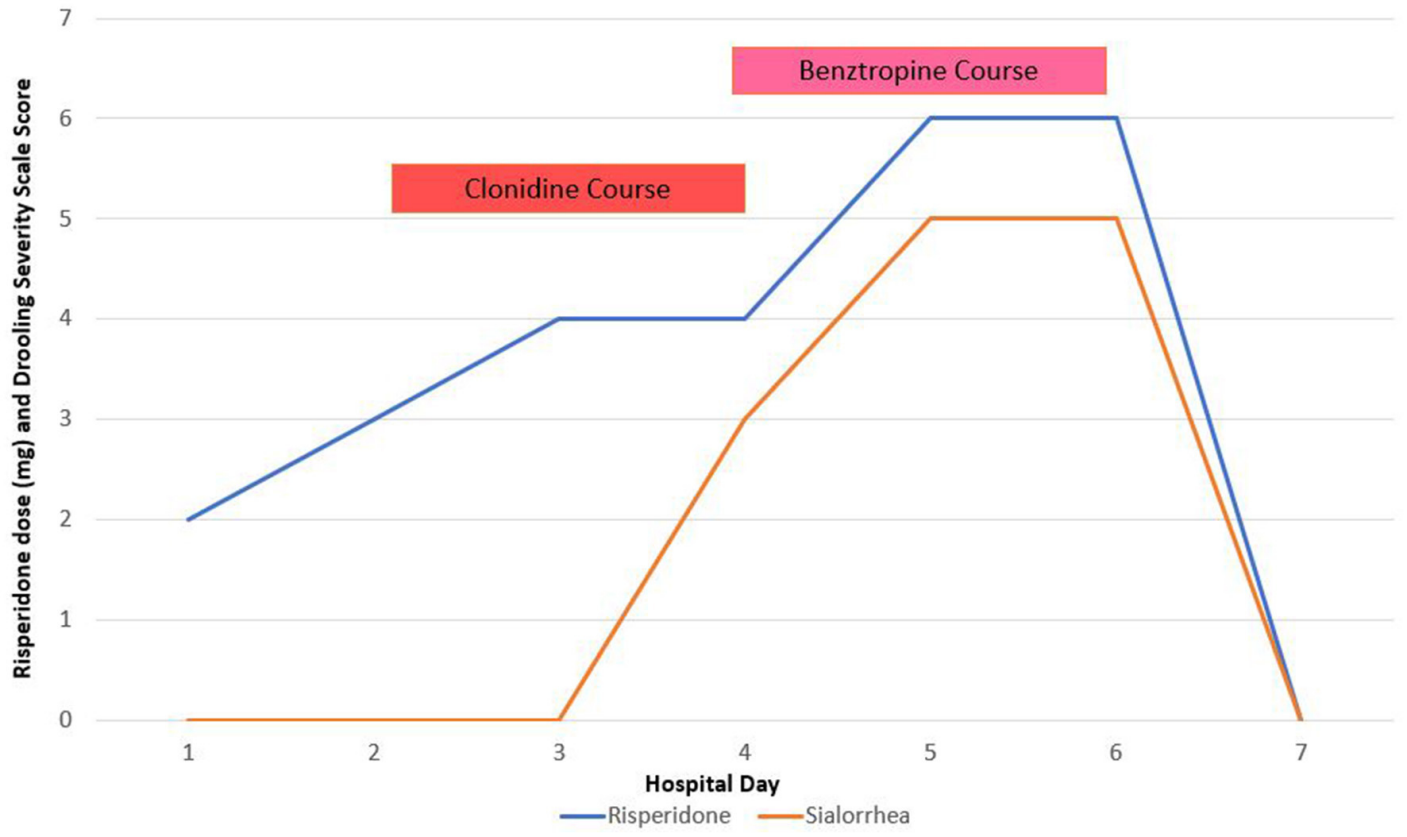
Medication course and adverse event timeline.

The next day, on hospital day 5, risperidone was also titrated to 6 mg daily. After the discontinuation of the clonidine, the patient had significantly worsened thick mucinous saliva and was found to have cogwheel rigidity with masked facies (Figure 1). In response, he started on a benztropine 2 mg daily but had no alleviation of severe mucinous sialorrhea and was wearing a towel over his shoulder due to the amount [Drooling severity scale rating: 5, profuse (2, 7, 8)]. The patient utilized atropine 1% ophthalmic drops (administered orally sublingually) but still had minimal relief of sialorrhea despite frequent use. His worsening rigidity and sialorrhea on hospital day 6 prompted medication changes, including discontinuing risperidone and benztropine and starting olanzapine 10 mg daily. After the medication changes, the patient's sialorrhea and rigidity began to subside and eventually resolved. The patient was later transitioned to quetiapine 300 mg prior to discharge and did not experience any further psychotropic-induced adverse reactions.

## Discussion

### Case analysis and scientific implications

This case report describes risperidone-induced sialorrhea unintentionally masked by simultaneous clonidine administration intended to treat hypertension. Interestingly, sialorrhea was present but mild when clonidine was present; however, when risperidone was further titrated and clonidine removed, significant worsening of sialorrhea developed, and this was scored by the drooling severity scale. There was no relief of sialorrhea with benztropine and minimal relief with atropine. Clonidine, an α_2_-adrenergic receptor agonist, did alleviate sialorrhea and suggests that risperidone's α_2_-adrenergic receptor antagonism is a key mechanism in risperidone-induced sialorrhea. We hypothesize that risperidone-induced sialorrhea has higher protein content (higher viscosity) compared to clozapine-induced sialorrhea due to risperidone's adrenergic stimulation mechanism. Although salivary composition analysis was not obtained in this patient, the physical examination did reveal congruent thick mucinous saliva. To investigate this hypothesis further, future cases that suspect risperidone-induced sialorrhea are suggested to obtain saliva composition analysis.

### Fundamentals of saliva production

**Figure 2:**
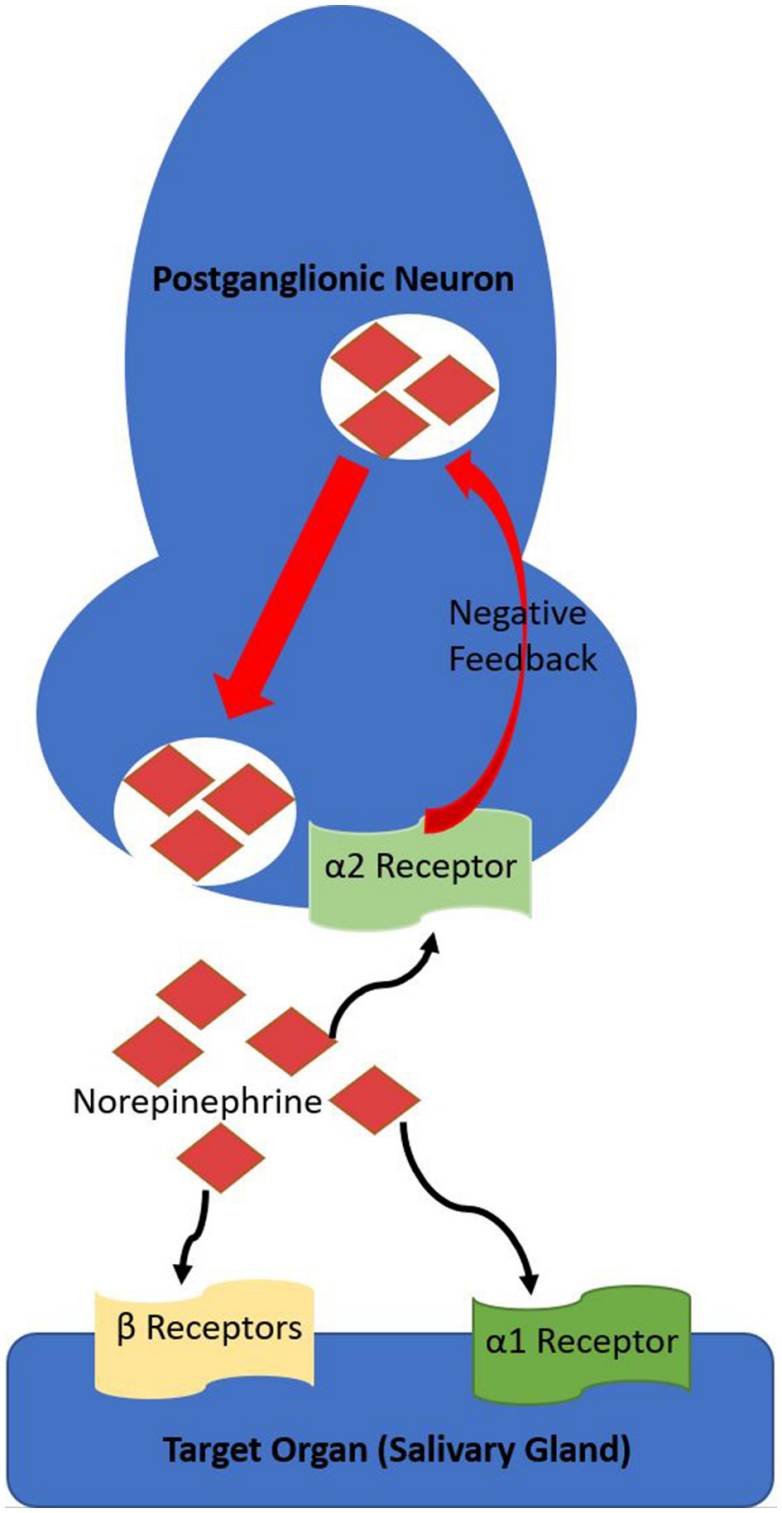
Overview of the sympathetic (adrenergic) pathway and α_2_-adrenergic receptor role in negative feedback. The α_2_-adrenergic receptor is a Gi-coupled metabotropic receptor, decreasing intracellular cAMP, thereby decreasing exocytosis.

Saliva production is a constant process, but the amount and viscoelasticity (protein content and viscosity) are dependent on multiple factors, including proportions of sympathetic vs. parasympathetic stimulation and circadian rhythm (1). Mastication induces parasympathetic stimulation, predominately by M_1_ and M_3_-Muscarinic receptors, which produces copious saliva of low protein concentration (low viscoelasticity) (1, 2). M_4_-muscarinic receptors stimulation also appears to increase saliva output (2, 9). Sympathetic (adrenergic) stimulation results in saliva production of lower volume but higher protein concentration (high viscoelasticity) (1, 2). α_2_-adrenergic receptors provide negative feedback for sympathetic stimulation (Figure 2), decreasing salivary flow when stimulated (10). Antagonism of α_2_-adrenergic receptors increases salivation by disinhibition, leaving α_1_-adrenergic and β-adrenoreceptors unopposed to induce a protein-rich, catecholamine-concentrated, and increased rate of flow hyper-salivatory state (10). The α_2_-adrenergic hypersalivation mechanism was demonstrated in multiple studies utilizing yohimbine, a potent α_2_-adrenergic antagonist (11, 12).

### Hypothesized pathophysiology of risperidone-induced sialorrhea

Risperidone and clozapine are both second-generation (atypical) antipsychotics but have differing neuroreceptor affinity across their mechanisms of action (Table 1); these differences are responsible for their differing therapeutic efficacies as well as their most common adverse reactions. We hypothesize that risperidone-induced sialorrhea is likely a result of potent α_2_-adrenergic antagonism. Risperidone-induced sialorrhea may have some pathophysiological contributions from its antimuscarinic activity; however, these neuroreceptor affinities are far less potent than its α_2_-adrenergic antagonism.

**Table 1:** Antipsychotic receptor binding affinities.

	**M_1_-muscarinic receptor**	**M_3_-muscarinic receptor**	**M_4_-muscarinic receptor**	**α_2_-adrenergic receptor**	**Dopamine D_2_ receptor**	**Serotonin 5-HT_2A_ receptor**
Risperidone	>10,000	>10,000	>10,000	8	3.8	0.15
Paliperidone	>10,000	>10,000	>10,000	80	2.8	1.2
Clozapine	1.4	109	27	158	210	2.59
Quetiapine	120	1,320	660	80	770	31

Data is represented by the equilibrium constant (K_i_), the nanomolar amount required to block 50% of the specified receptor. Pharmacokinetically, the lower an equilibrium constant (K_i_), the stronger a receptor is bound (2, 13, 14).

Clozapine-induced sialorrhea is much more common than risperidone-induced sialorrhea, and its pathophysiology is more robustly studied. Clozapine-induced sialorrhea is a result of clozapine's potent M_4_-muscarinic partial agonism and its metabolite norclozapine having potent muscarinic agonism (15). Clozapine-induced sialorrhea likely has some pathophysiological contribution from α_2_-adrenergic antagonism, but it is not as potent at this neuroreceptor in comparison to M_3_ and M_4_ muscarinic receptors or to risperidone. This likely results in lower viscoelastic saliva compared to risperidone-induced sialorrhea, but no direct comparisons are documented, and it also probably slightly varies from case to case. An additional pathophysiological mechanism of sialorrhea from clozapine results from decreased laryngeal peristalsis and inhibition of swallow reflex from its potent muscarinic modulation (16), which is likely less contributory in risperidone-induced sialorrhea.

### Management of risperidone-induced sialorrhea

Sialorrhea is uncomfortable for the individual experiencing it and has significant psychosocial implications. Wet clothing may result in social stigmatization and embarrassment. The need to carry towels and “spit cups” can impair vocational functioning (2). The psychosocial complications of antipsychotic-induced sialorrhea may be a reason for a patient to self-discontinue their medication treatment (17, 18). Therefore, it is paramount to manage this adverse reaction in cases the therapeutic benefit of risperidone is substantial. Further, managing sialorrhea reduces the risk of its medical complications, which can include choking sensation, dysphonia, irritated, and macerated skin in perioral areas, cheilitis, sleep disturbance, and aspiration (2).

Switching antipsychotics or reducing the dose of risperidone would be an optimal first step in managing suspected risperidone-induced sialorrhea. If risperidone-induced sialorrhea only occurs after titrating risperidone to a high dose, tapering down to a previously tolerated lower dose may be considered (3, 4), although this will likely congruently decrease its antipsychotic effectiveness. Many of the therapeutic benefits of risperidone are also due to its conversion to its active metabolite of paliperidone. Paliperidone has a 10-fold weaker affinity for α_2_-adrenergic antagonism compared to risperidone (Table 2). Therefore, patients who benefit from the antipsychotic effects of risperidone but develop mild-moderate sialorrhea on it may be able to maintain similar treatment efficacy by transitioning to paliperidone and monitoring for a reduction in sialorrhea. However, paliperidone-induced sialorrhea has also been reported (19). Risperidone metabolism utilizes cytochrome P450 enzymes 2D6 and 3A4, and genetically inherited impaired activity of these enzymes may lead to the predisposition of risperidone-induced adverse reactions, including sialorrhea (20).

**Table 2:** Summary of salivary flow by the agonism or antagonism associated with common antipsychotic mechanisms of action.

	**Saliva flow modulation by receptors of interest**			
**Receptor**	**M1-muscarinic receptor**	**M**_3_ **-muscarinic receptor**	**M**_4_ **-muscarinic receptor**	α_2_**-adrenergic receptor**
Agonism	Increased flow	Increased flow	Increased flow	Decreased flow
Antagonism	Decreased flow	Decreased flow	Decreased flow	Increased flow (increased viscoelasticity)

Viscoelasticity describes protein concentration in saliva (1, 2, 9, 10).

As described in this case report, clonidine has been reported as alleviating risperidone-induced sialorrhea, hypothesized due to its properties as a centrally acting α_2_-adrenergic receptor agonist. Similarly, Gajwani et al. (5) also reported a case of risperidone-induced sialorrhea that did not respond to benztropine but was alleviated with clonidine. Other medications in this class include lofexidine, guanfacine, guanabenz, α-methyldopa, and moxonidine, but have not been reported in the literature as an attempted treatment for risperidone-induced sialorrhea (2). β-adrenergic receptor antagonists such as propranolol are theoretically another treatment option for risperidone-induced sialorrhea. This treatment would aim to halt the unopposed β-adrenergic receptors resulting from risperidone's α_2_-adrenergic antagonism (Figure 2) (2). β-adrenergic receptor antagonists are known to decrease salivary viscoelasticity but not necessarily the volume of saliva (21).

Anticholinergic (antimuscarinic) medications are commonly prescribed medications for treating clozapine-induced sialorrhea. Atropine ophthalmic drops are frequently used and administered sublingually and on the inside of the cheeks, with many patients reporting relief of sialorrhea (22). We predict that due to likely differences in pathophysiology for clozapine vs. risperidone-induced sialorrhea, selective and non-selective anticholinergic (antimuscarinic) medications are less likely to relieve risperidone-induced sialorrhea, despite some efficacies reported with clozapine-induced sialorrhea. Still, risperidone dose reduction and the addition of diphenhydramine were reported as therapeutic in one case of risperidone-induced sialorrhea (6); and another case was treated with risperidone dose reduction down to a previously tolerated dose and adding biperiden (4).

## Conclusions

The pathophysiology of antipsychotic-induced sialorrhea is complex and varies between antipsychotics. Risperidone-induced sialorrhea is suspected of having prominent adrenergic pathophysiology, likely composed of highly viscoelastic saliva (high protein content), differing from the more commonly encountered clozapine-induced sialorrhea. Risperidone-induced sialorrhea is reported to be more likely to respond to dose reduction, treatment with α_2_-adrenergic receptor agonists or β-adrenergic receptor antagonists, and less likely to respond to anticholinergic (antimuscarinic) medications.

## Data availability statement

The original contributions presented in the study are included in the article/supplementary material, further inquiries can be directed to the corresponding author.

## Ethics statement

The studies involving human participants were reviewed and approved by Kern Medical Institutional Review Board. The patients/participants provided their written informed consent to participate in this study. Written informed consent was obtained from the participant/patient(s) for the publication of this case report.

## Author contributions

TT and AK formulated the analysis and wrote the manuscript. All authors contributed to the article and approved the submitted version.
